# Supplementary material for: Molecular Insights into Intramuscular Unsaturated Fatty Acid Deposition in Lambs Through Multi-Omics Profiling
Source: Animals (Basel). 2025 Sep 6;15(17):2617. doi: 10.3390/ani15172617 (PMC12427329; doi:10.3390/ani15172617)
Supplement: Supplementary file 1 [file animals-15-02617-s001.zip › animals-3833079-supplementary.pdf]

Supplementary Table S1. Primer sequences used for RT-qPCR

Target Gene	Primer Sequence (5' → 3')	Product Size (bp)	Annealing Temperature (°C)
MYH7	F: GAACAGGCCAACACCAACCT<br>R: CCTCATTCAAGCCCTTGGCG	145	60
FADD	F: TCCCCTGAGGACGGAAAGAA<br>R: GGAGACGGTGACCCTTCATC	109	60
ABCB11	F: CGACAACGGTCCAAGTCTCA<br>R: CCCATGAGCCATTGGTGTCT	116	60
CSNK2A2	F: TGAGAAGTCCCGTGGTGAGA<br>R: GAGTGAGCTGCAAGCAAACC	72	60
CTSB	F: CCAGGGTTGTGTGTCACTGT<br>R: GGCACCCAGATTCATGGGAA	89	60
β-actin	F: TTCTAGGCGGACTGTTAG<br>R: TGCCAATCTCATCTCGTT	84	60

Supplementary Table S2. Information of primary antibodies used in this study

Primary Antibody	Host	Dilution	Supplier & Catalog ID
MYH7	Rabbit	1:200	Beijing Bioss, bs-9862R
FADD	Rabbit	1:300	Beijing Bioss, bs-0511R
ABCB11	Rabbit	1:300	Beijing Bioss, bs-42133R
CSNK2A2	Rabbit	1:300	Proteintech, 10606-1-AP
CTSB	Rabbit	1:200	Beijing Bioss, bs-1500R
β-actin	Rabbit	1:200	Beijing Bioss, bs-0061R
slow MYHC	Mouse	1:1000	Sigma, M8421
fast MYHC	Mouse	1:1000	Sigma, M4276
